# Cortical Fenestration for Megaprosthesis Stem Revision

**DOI:** 10.2174/1874325001711010234

**Published:** 2017-03-31

**Authors:** Vincent Y. Ng, Philip Louie, Stephanie Punt, Ernest U. Conrad III.

**Affiliations:** 1 University of Maryland Medical Center, Department of Orthopedics, Baltimore MD, USA; 2 Rush University, Chicago IL, USA; 3 University of Washington Medical Center, Department of Orthopaedics and Seattle Children's Hospital, Seattle WA, USA

**Keywords:** Stem fracture, Cortical fenestration, Window, Revision, Megaprosthesis, Tumor

## Abstract

**Background:**

The most common modes of failure for megaprostheses are aseptic loosening followed by periprosthetic infection and stem fracture. Surgical technique for bone and implant exposure is controversial and may influence the success of revision and the need for additional future revisions. The purpose of this study was to evaluate the effectiveness of cortical fenestration for megaprosthesis revision, particularly for stem fracture.

**Methods:**

From 1985-2014, 196 adult and pediatric patients underwent limb salvage with a distal femoral or proximal tibial megaprosthesis (109 cemented, 87 pressfit). A retrospective chart review was performed to assess the rate of revision based on cemented or pressfit fixation and the use of a cortical window to extract the failed stem.

Results:

27% (29 of 109) of cemented and 18% (16 of 87) of pressfit implants were revised for stem failure. The reasons for revision in the cemented group were loosening (62%), infection (24%), and stem fracture (13%). In the pressfit group, the reasons were loosening (43%), infection (31%), stem fracture (6%), limb-length discrepancy (6%), malrotation (6%), and local recurrence (6%). A cortical window was used in 10 of 45 initial revisions (7 cemented, 3 pressfit) including all of the stem fractures, and in 2 of 15 subsequent re-revisions.

**Conclusion:**

Cortical fenestration is an effective, bone-preserving method of implant extraction, particularly for broken or cemented stems. It is associated with low rates of loosening and no increase in periprosthetic fractures.

## INTRODUCTION

Stem fracture is an uncommon but distinct mode of failure for oncologic implants. It is reported in the arthroplasty literature that cantilever bending forces can lead to metal fatigue and stem fracture [1-5]. This type of loading can be observed with distal femoral and proximal tibial megaprostheses, especially if osseous resorption or stress shielding occur at the level of the initial osteotomy and implant collar.

Removal of a well-fixed cemented or pressfit stem that is broken within the canal can be challenging. Standard revision techniques using gouges and flexible osteotomes are typically ineffective. Overreaming the stem with trephines is often unsuccessful for cemented or for bowed stems and are excessively destructive to the remaining cortical bone. Performing an osteotomy along the length of the incarcerated stem may create a cortical fracture. 

Varying descriptions for cortical fenestration for removal of hip stems have been published [5-9]. The objectives of this study are to 1) report a series of fractured megaprostheses stems about the knee, 2) describe an effective technique for stem removal, and 3) evaluate the effectiveness of this technique for varying revision situations.

## METHODS AND MATERIALS

A retrospective chart review was performed for a single surgeon (EUC) from 1985-2013. 330 adult and pediatric patients were identified as having undergone limb salvage with a megaprosthesis, of which 196 (109 cemented, 87 pressfit) involved the distal femur or proximal tibia. There were 45 revisions. Case details including method of fixation for the primary prostheses, reason for implant failure, and method of stem extraction were recorded. All data were collected in accordance to IRB.

**Fig. (1):**
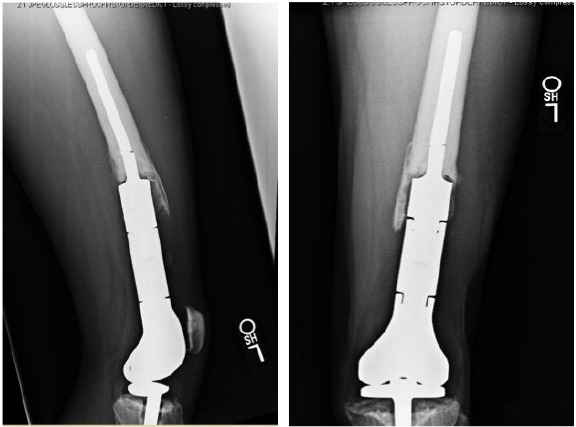
Lateral and anteroposterior radiographs of a broken stem.

Cortical fenestration was performed in all cases with a fractured stem (Fig. **1**) and in select difficult revisions for other etiologies for a total of 12. Using intraoperative fluoroscopy, the level of the cortical window was planned such that a 4 cm or greater length of circumferential intact cortical bone would remain at the end of the bone adjacent to the stem collar. The cortical window would extend in length to near the tip of the stem. A high-speed pneumatic drill with a fine tip was used to perforate the cortex at 5 mm intervals and outline a long oval cortical window. For the femur, the window was placed anteriorly. For the tibia, the window was placed anteromedially or anterolaterally. To avoid compromising the cortical integrity of more than one side of the bone, the width of the window was limited to the width of the intramedullary canal. An approximately 10-20 mm osteotome was used to carefully complete the window.

Once the window was removed, a high-speed burr with a wire-passer tip was used to remove any easily visible cement Fig. (**2**). An ultrasonic cement removal device such as the Ultra- Drive^®^ (Biomet, Warsaw, IN) or OSCAR^®^ (Orthosonics, Chatham, NJ) was used to remove cement from the sides of the implant and within the canal. A burr was used in the window to create a transverse divot in the stem such that an osteotome could be placed into the divot and the entire stem be driven retrograde out of the canal using a mallet and the osteotome. After the stem was removed, the remaining cement was removed with the ultrasonic device. It should be noted that the ultrasonic device was also effective at disrupting osseous ingrowth fixation for pressfit stems.

A long pressfit stem that bypassed the cortical window by at least 4 cm was typically used for reconstruction. The cortical window was fixed with two cerclage cables prior to placement of the final stem Fig. (**3**). If the new implant was cemented, bone wax was placed around the edges of the window to prevent cement extravasation. The collar of the new stem was seated into cortical bone to minimize a “flagpole” phenomenon and bending forces on the stem.

**Fig. (4):**
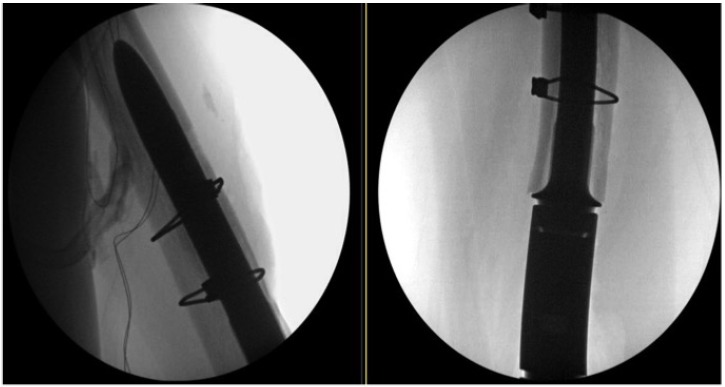
Lateral and Anteroposterior Radiographs Postoperatively.

Postoperatively, patients were kept partial weight-bearing for 6 weeks if no cortical fenestration was needed and 12 weeks if cortical fenestration had been performed. Postoperative radiographs were obtained to document the integrity of the cortical window (Fig. **4**).

## RESULTS

27% (29 of 109) of cemented and 18% (16 of 87) of pressfit prostheses were revised for stem failure. The reasons for revision in the cemented group were loosening (62%), infection (24%), and stem fracture (13%). In the pressfit group, the reasons were loosening (43%), infection (31%), stem fracture (6%), limb-length discrepancy (6%), malrotation (6%), and local recurrence (6%). A cortical window was used in 10 of 45 initial revisions (7 cemented, 3 pressfit) including all of the stem fractures, and in 2 of 15 subsequent re-revisions.

17% (2 of 12) revision cases in which a cortical window was used required subsequent re-revision for infection (8%) and for loosening (8%). Rerevision was necessary in 33% (16 of 48) cases in which no cortical window was used to remove the failed stem. The reasons for re revision in the cases without a cortical window were loosening (25%; 12 of 48) and infection (8%; 4 of 48).

## DISCUSSION

Stem fracture due to cantilever bending and inadequate proximal bone support is a well- known phenomenon in total hip arthroplasty, but it is less studied in megaprostheses around the knee. Improved manufacturing technology and larger stem diameter may reduce the rate of this complication. It can be challenging to remove a wellfixed or wellcemented fractured stem while preserving the remaining bone stock for further reconstruction. If the stem is bowed, conventional techniques such as hollow trephine reamers or flexible osteotomes are not reliable. If the stem is broken, it is typically countersunk within the diaphysis, retrograde techniques are ineffective, and some type of cortical fenestration is required for exposure.

In this series of 40 revision cases spanning more than three decades, stem fracture was responsible for approximately 10% of stem revision cases. This is lower than the 24% for distal femoral and proximal tibial replacements reported by Schwartz *et al.* [10].

The cortical fenestration technique described here provides a consistent, reproducible method to remove the stem and allow for reliable placement of a competent stem. It is associated with low rates of subsequent loosening and no increase in periprosthetic fractures.

## Figures and Tables

**Fig. (2):**
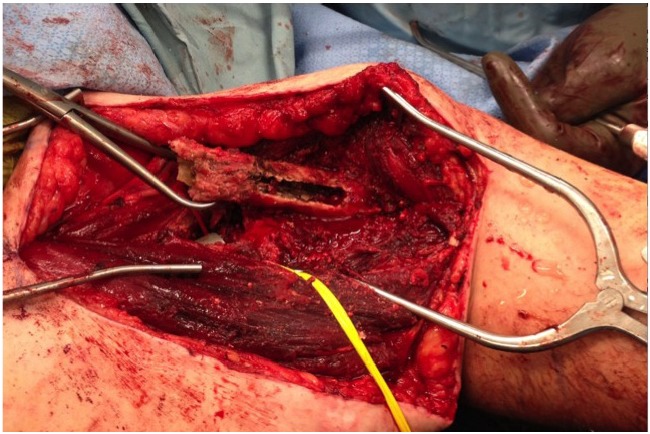
Intraoperative photo of the cortical window after extraction of a broken stem.

**Fig. (3):**
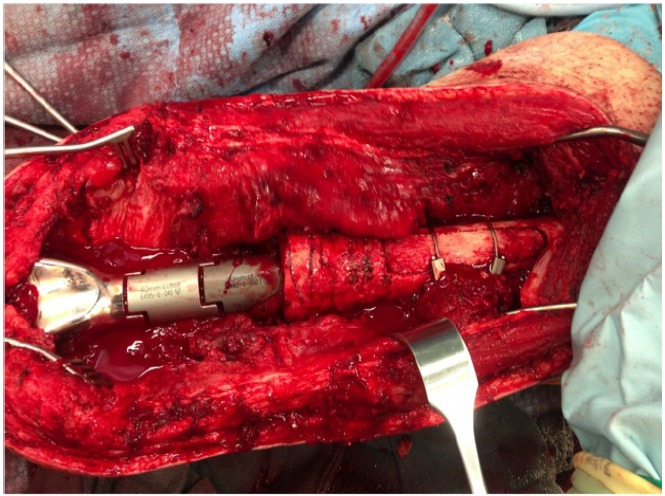
Intraoperative photo of revision components with refixation of cortical window.
